# PIEZO Force Sensing in Vascular Biology: An Explosion of New Knowledge, Concepts and Opportunity

**DOI:** 10.1002/advs.202511774

**Published:** 2025-08-24

**Authors:** David J Beech

**Affiliations:** ^1^ Leeds Institute of Cardiovascular and Metabolic Medicine School of Medicine University of Leeds Leeds LS2 9JT UK

**Keywords:** calcium signalling, cardiovascular, endothelium, mechanical force, nonselective cation channel, shear stress, stretch

## Abstract

The discovery of PIEZO1 channels as robust, rapidly activating, electrically transducing mechanical force sensors of endothelial biology led to intense research efforts that have transformed the appreciation and understanding of pivotal relationships between mechanical forces and cardiovascular health. This narrative review of the scientific literature highlights discoveries about the PIEZOs, PIEZO1 and PIEZO2, in vascular biology from the embryo to adult stages including in vascular and valve formations, vascular expansion and arrest, lymphatic and venous integrity, blood pressure regulation, the aortic baroreceptor reflex, microvascular density for muscle and physical exercise capabilities and the regulation of lipid homeostasis, cerebral hyperemia and leukocyte extravasation. Concepts are discussed for how the channels work at the molecular level and integrate with other cell components and how they signal downstream for appropriate tissue responses. A PIEZO‐centric hypothesis is debated for the core fluid flow sensing property of cardiovascular biology. PIEZO contributions to vascular and vascular related disease problems are discussed. In summary, this is an exciting area of research that is revolutionizing the understanding of the cardiovascular system and revealing new ways to address unsolved cardiovascular disease.

## Graphical abstract

Knowledge of the remarkable mechanical force sensing and electrically transducing PIEZO1 and PIEZO2 channels is discussed across vascular biology and its cell types from the embryonic to adult stages in health, disease and old age. How the channels work, relate to other factors and signal for tissue responses to mechanical forces is debated. Ideas for future research are suggested.

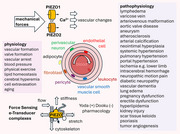

## 1 Introduction

The discovery of PIEZO channels^[^ ^1^ ^]^ led to powerful evidence for mechanical force sensing as a pivotal factor in many aspects of vascular biology, the full extent of which is probably only just starting to be appreciated. Here in this narrative review of the scientific literature since the PIEZO1 channel was first reported as an endothelial fluid flow sensor determining vascular architecture,^[^ ^2^, ^3^ ^]^ key points are highlighted and debated from the substantial knowledge now available. The scope of the review across vascular biology extends from endothelial cells and vascular smooth muscle cells to the perivascular cells and neurons as well as blood cells interacting with the vascular wall. It outlines physiological roles of PIEZOs from developmental to adult biology, in pregnancy, blood pressure regulation, physical exercise, lipid homeostasis, bone strength, somatosensory stimulation, tissue scarring, immune response, bacterial resilience and aging. It considers the properties of the channels, how the channels work as molecular entities, factors that modulate the channels and integrate them into cells and tissues, and the downstream signalling principles of the channels. It explores the idea that PIEZOs are pivotal force sensors of endothelial flow sensing, addressing their relationships to other cell components and other proposed sensors. It considers roles of PIEZOs in pathophysiology as determined by studies of the genetics of natural human disease and disease induced artificially in animals. It discusses the potential for therapeutic targeting of PIEZOs and summarizes key concepts and questions for future research.

## 2 Embryonic, Fetal and Postnatal Development

### 2.1 Vascular Remodeling for Embryonic Growth

A key stimulus for embryonic vascular expansion and thus organ development is the heart's pumping of blood through the nascent endothelial tubes, starting at about embryonic day 8.5 (E8.5) in laboratory mice.^[^ ^4^ ^]^ Therefore, the finding that homozygous *Piezo1* gene‐disrupted mice (i.e., global PIEZO1 knockouts) suffered embryonic growth retardation and lethality from E9.5 despite a normal heart beat was consistent with PIEZO1 having a role in fluid flow‐evoked vascular maturation.^[^ ^2^, ^3^ ^]^ In support of this idea, imaging of the embryo's yolk sac revealed a failure of vascular remodeling that is normally required for vascular maturation and embryo progression.^[^ ^2^, ^3^ ^]^ Endothelial cells isolated from the knockout embryos did not remodel in the direction of fluid flow or show fluid flow‐evoked intracellular calcium ion (Ca^2+^) elevations, consistent with the embryonic effects of PIEZO1 knockout originating in altered endothelial cell properties.^[^ ^2^ ^]^

### 2.2 Lymphatic Formation

The growth‐restricted embryos of *Piezo1*‐disrupted mice were found to have pericardial effusion, an abnormal retention of fluid around the heart.^[^ ^3^ ^]^ Similar observations were made in PIEZO1‐depleted zebrafish.^[^ ^5^ ^]^ Therefore, PIEZO1 is important in the physiological control of pericardial fluid volume, reflecting its more general role in lymphatic endothelium and the formation of lymphatic valves and thus tissue drainage.^[^ ^6^, ^7^ ^]^ In support of this idea, disruptive *PIEZO1* variants in people have been found to associate with non‐immune fetal hydrops (NIFH)^[^ ^8^ ^]^ as discussed in the pathophysiology section of this article. Moreover, an endothelial *Piezo1*‐disrupted mouse strain that survives to birth revealed a postnatal lymphatic phenotype, with the mice dying shortly after birth from generalized lymphedema with pleural effusion.^[^ ^6^ ^]^ Variation in the timing of the lethality from mid‐gestation^[^ ^2^ ^]^ to shortly after birth^[^ ^6^ ^]^ may reflect an experimental dependence on the embryonic timing of the activation of the endothelial promoter (i.e., *Tie2*) used to drive gene disruption.^[^ ^9^ ^]^

### 2.3 Aortic Valve Formation

Embryonic *Piezo1* expression was noted in endothelium of the ventricular outflow tract and atrioventricular canal,^[^ ^3^ ^]^ sites where blood flow determines the formation of the aortic valve. The role of PIEZO1 in valve formation was then studied in zebrafish where the valve can be visualized experimentally in the intact animal. *Piezo1*‐disrupted zebrafish showed disturbed outflow tract valve morphogenesis, indicating a requirement for PIEZO1 in valve formation.^[^ ^10^ ^]^ PIEZO1 was suggested to participate in valve formation by mediating fluid flow sensing in the endothelial cell layer and a stretch effect in the smooth muscle cell layer. *Piezo2* gene disruption similarly disturbed valve formation, albeit less effectively, and a combination of *Piezo1* and *Piezo2* disruption almost prevented valve formation.^[^ ^10^ ^]^ In a separate zebrafish study in which PIEZO1 was depleted, and thus reduced in abundance rather than negated, there was similarly disturbed outflow tract and valve formation.^[^ ^5^ ^]^

### 2.4 Coronary Vasculature Formation

Genetically engineered tracking of *Piezo2* gene transcription in mice has led to the suggestion of PIEZO2 expression in cells of the sinus venosus and coronary plexus at E11.5–13.5.^[^ ^11^ ^]^ From E15.5 to postnatal day 2, expression was mainly in the coronary endothelium of capillaries, with little expression in other cell types of the heart up to early adult stage.^[^ ^11^ ^]^ PIEZO2‐like mechanically activated currents were observed in endothelial cells at E13.5–18.5.^[^ ^11^ ^]^ In *Piezo2*‐disrupted hearts, the branching of the left coronary artery into the circumflex artery and the left anterior descending artery did not follow the normal pattern, and coronary vessels were constricted.^[^ ^11^ ^]^ In contrast to *Piezo2* expression, there was *Piezo1* expression at all developmental and adult stages.^[^ ^11^ ^]^ Three different mechanically activated currents were observed in E13.5–18.5 endothelial cells of the yolk sac, suggesting that PIEZO2 channels occurred alongside other mechanically activated channels such as PIEZO1 channels.^[^ ^11^ ^]^

### 2.5 Brain Vascular Pattern Formation

Zebrafish studies have also shown that PIEZO1 is required for endothelial tip cell intracellular Ca^2+^ events that determine vascular branching patterns in the brain depending on their frequency.^[^ ^12^ ^]^ Mechanical stretch and tissue stiffness mimicked by a micropipette were found to evoke PIEZO1‐dependent Ca^2+^ events in the tip cells, with more force causing more Ca^2+^ events and tip retraction.^[^ ^12^ ^]^ PIEZO1‐deleted larvae of the fish showed increased complexity of brain vasculature, suggesting dysregulated vascular patterning.^[^ ^12^ ^]^

### 2.6 Mural Layer Formation

In major parts of the vasculature, there is accumulation of mural cells such as pericytes and vascular smooth muscle cells around the endothelial tubes depending on blood flow characteristics.^[^ ^13^, ^14^ ^]^ In zebrafish, global and endothelial‐specific *Piezo1* disruption reduced pericyte proliferation and pericyte coverage of developing blood vessels.^[^ ^14^ ^]^ Global *Piezo1* and *Piezo2* disruption decreased vascular smooth muscle cell accumulation in the dorsal aorta.^[^ ^13^ ^]^ In mice, endothelial *Piezo1* disruption caused E12.5–13.5 embryos to be paler (less red), suggesting a reduced vascular density^[^ ^13^ ^]^ consistent with previous observations^[^ ^2^ ^]^ but there was also reduced vascular smooth muscle thickness and cell number around the dorsal aorta that suggested endothelial PIEZO1 regulated the smooth muscle and pericyte layers.^[^ ^13^ ^]^

### 2.7 Placental Vasculature

Cells commonly used in the laboratory to reveal generic endothelial cell properties are the human umbilical vein endothelial cells (HUVECs) because they are quite easily obtained in large numbers from routinely discarded human tissue and retain in cell culture key characteristics of native endothelial cells such as a sensitivity to the force of shear stress caused by fluid flow. These cells are also specifically relevant to the umbilical vein that develops from the yolk sac and critically conveys oxygenated blood from the placenta to the fetus. Studies of these cells first revealed that shear stress rapidly activates a Ca^2+^ permeable nonselective cation channel.^[^ ^15^ ^]^ The unitary conductance of this channel did not match that of other candidates for the shear stress sensor such as P2X4, TRPV4, TRPC1 and polycystin‐2 (PKD2) channels but rather that of the mechanically activated PIEZO1 channel identified in a neuroblastoma cell line.^[^ ^1^, ^2^, ^16^ ^]^ Along with other observations, this finding led to the suggestion that the HUVEC shear stress‐activated channel^[^ ^15^ ^]^ is a vascular form of the PIEZO1 channel.^[^ ^2^, ^3^ ^]^ Fetoplacental microvascular endothelial cells isolated from placentas of normal human pregnancies also failed to remodel to the direction of fluid flow without PIEZO1, and endothelial cells freshly isolated from placental artery displayed PIEZO1‐like channel activity in response to shear stress.^[^ ^17^ ^]^ Therefore, PIEZO1 might also be important in the formation and health of placental microvasculature and conduit vessels.

### 2.8 Postnatal Vascular Expansion

Regions of the nervous system including the retina and cerebellum undergo postnatal vascular expansion. Rather than endothelial PIEZO1 playing a role, PIEZO2 predominates here in perivascular neurons that create contacts with endothelial cells, driving the growth of penetrating vessels.^[^ ^18^ ^]^ Deletion of this PIEZO2 has led to retinal vascular perfusion deficits, tissue hypoxia, enhanced susceptibility to ischemic ocular insults and abnormal cerebellar vascular patterning.^[^ ^18^ ^]^ PIEZO2‐expressing sensory neurons were also found to be associated with the developing renal vasculature^[^ ^19^ ^]^ and so PIEZO2 might contribute similarly in the kidneys where there is also postnatal vascular expansion.^[^ ^20^ ^]^

### 2.9 Postnatal Vascular Arrest

Bone is a vascularized organ that, during postnatal growth, contains angiogenic type H vessels characterized by the high (H) expression of PECAM1 and endomucin at sites of active bone growth.^[^ ^21^, ^22^ ^]^ The vessels associate with osteoblasts, the mechanical stimulation of which by body weight activates PIEZO1 to release an extracellular matrix protein to switch the vessels from H to L (low) type, thereby inhibiting bone growth.^[^ ^22^ ^]^

## 3 Adult Physiology

The developmental roles of PIEZOs outlined above have consequences for early life and often adult life, for example through altered lymphatic and cardiac valve physiology, but PIEZOs also have other roles that may only be apparent at the adult stage.

### 3.1 Endothelial Regulation of Blood Pressure

Adult roles of endothelial PIEZO1 can be studied in mice by conditionally disrupting endothelial *Piezo1* after vascular development. In this way, the disruption of endothelial PIEZO1 has been found to increase systolic blood pressure or have no effect on diastolic or systolic blood pressure^[^ ^23^, ^24^ ^]^ or conversely decrease blood pressure during running wheel activity.^[^ ^23^ ^]^ These varied effects are not yet fully understood, although they may be explained by vascular bed diversity, blood flow redistribution in whole‐body physical activity, complexity in the molecular mechanisms of endothelium‐dependent modulation of vascular tone due to nitric oxide (NO) and membrane potential‐dependent mechanisms^[^ ^23^, ^24^ ^]^ and multisystem regulation of blood pressure involving also the kidneys, for example. Despite the challenges of understanding the net effects on blood pressure, the whole‐body observations have indicated the importance of considering physical activity when investigating PIEZO channels.

### 3.2 Endothelial and Perivascular Contributions to Whole‐Body Physical Capability

2 weeks after endothelial *Piezo1* disruption in adult mice, transient reductions were observed in physical performance^[^ ^23^ ^]^ but 10 weeks after disruption, stronger, sustained effects were observed that included the halving of running speed and the halving of distance run without alterations in the attempts to run, suggesting reduced physical capability but retained desire to run.^[^ ^25^ ^]^ Associated with these effects, there was reduced skeletal muscle capillary density, revealing a role of endothelial PIEZO1 in maintaining the vitality and thus structural integrity of the muscle microvasculature and, in this way, being important for physical exercise capability.^[^ ^25^ ^]^ In a separate study, genetic disruption of PIEZO1's positive regulation by protein kinase A reduced the performance of mice in a rotating rod endurance test.^[^ ^26^ ^]^ Although this protein kinase A‐focused study was not endothelial specific, evidence was provided that the genetic disruption had prominent endothelial consequences.^[^ ^26^ ^]^ In zebrafish studies, body motion was observed to trigger intracellular Ca^2+^ events in endothelial cells.^[^ ^27^ ^]^ When the fish performed an escape tail flick, Ca^2+^ events occurred in endothelial cells of arteries.^[^ ^27^ ^]^ These motion‐triggered endothelial Ca^2+^ events depended on PIEZO1 and mechanical forces in the muscle rather than the heartbeat.^[^ ^27^ ^]^ In separate studies in mice, voluntary running promoted the expansion of peri‐arteriolar niches in load‐bearing bones; lymphoid progenitors associated with arterioles were reduced after *Piezo1* disruption in bone marrow stromal cells, suggesting importance of force sensing by PIEZO1 in the maintenance of bone health, including in the clearance of infection from bone.^[^ ^28^ ^]^ Taken together, these studies suggest that endothelial PIEZO1 channels have important roles in whole‐body physical activity and health benefits of exercise.

### 3.3 Endothelial Regulation of Lipid Homeostasis

In whole‐body physical activity, redistribution of blood to muscle is enabled by a reduction of blood flow to other organs such as the liver. Mediated by endothelial PIEZO1, reduced portal flow was found to increase specific gene expression in hepatocytes, the key parenchymal cell of the liver, regulating cholesterol and bile homeostasis.^[^ ^29^ ^]^ A similar endothelial regulation of parenchymal cells was indicated in the small intestine, in this case controlling lipid excretion.^[^ ^29^ ^]^ Therefore, endothelial PIEZO1 provides a connection between fluid flow at the endothelium and the regulation of parenchymal cells that participate in whole‐body lipid homeostasis.

### 3.4 Endothelial Regulation of Brain Vascular Structure and Hyperemia

In whole‐body physical activity, cerebral blood flow is usually maintained relatively constant but there are other important regulations of the brain's circulation. In middle cerebral artery of adult mice heterozygous for global *Piezo1* disruption, the normal alignment of endothelial cells to the direction of blood flow was disturbed, suggesting expression and functional relevance of PIEZO1 in endothelium of the cerebral circulation.^[^ ^2^ ^]^ Consistent with this suggestion, PIEZO1 single channel events were detected in retinal vascular endothelial cells.^[^ ^30^ ^]^ Moreover, endothelial *Piezo1* disruption in mice enhanced cerebral functional hyperemia caused by whisker stimulation and slowed the recovery from hyperemia, which suggests that endothelial PIEZO1 counteracts hyperemia and facilitates recovery of normal blood flow after somatosensory stimulation.^[^ ^31^ ^]^

### 3.5 Blood Pressure Sensing in the Aortic Arch

Physiological regulation of blood pressure and heart rate importantly involves the baroreceptor reflex mediated by pressure sensors in the aortic arch. Retrograde labelling of carotid sensory neurons in mice has suggested that cells forming baroreceptors express *Piezo1* or *Piezo2* mRNA.^[^ ^32^ ^]^ *Piezo1* and *Piezo2* gene disruption (i.e., PIEZO1 and PIEZO2 double knockout) in nodose and petrosal ganglion neurons that contribute the sensory neurons of the baroreceptor reflex was found to prevent the normal baroreceptor reflex that decreases heart rate in response to increased systolic blood pressure, while *Piezo1* or *Piezo2* disruption alone had no effect.^[^ ^32^ ^]^ Double knockout prevented the aortic depressor nerve activity that normally occurs with elevated blood pressure as well as the heart rate increase caused by blood pressure lowering.^[^ ^32^ ^]^ The double knockout also increased blood pressure variability,^[^ ^32^ ^]^ as expected when there is impaired baroreceptor function. Direct stimulation of PIEZO2‐positive baroreceptor afferents, achieved through engineered optical control, lowered blood pressure.^[^ ^32^ ^]^ Therefore, the two PIEZO types work together to enable physiological pressure sensing in the aortic arch, coupling heart rate to blood pressure. Consistent with these ideas and a suggested dominance of PIEZO2 in this system, other studies showed that activation of PIEZO2‐positive nodose‐petrosal‐jugular neurons decreased heart rate in mice, and that ablation of the PIEZO2 neurons eliminated the baroreceptor reflex.^[^ ^33^ ^]^ PIEZO2‐positive nerve terminals have also been seen to form claw‐like structures along the outer edge of the smooth muscle layer of the aortic arch, neatly positioning PIEZO2 to detect increases in arterial diameter with each pressure pulse.^[^ ^33^ ^]^

### 3.6 Endothelial, Leukocyte Extravasation and Immune Response in Tissue Injury

Endothelial PIEZO1's roles in vascular development and its participation in phenomena such the migration of endothelial cells in culture^[^ ^2^ ^]^ have suggested that endothelial PIEZO1 might also contribute in physiological responses to tissue injury and associated repair processes. Consistent with this idea, disruption of endothelial *Piezo1* in mice was found to slow skin wound closure after injury and slow recovery from hind limb ischemia^[^ ^34^ ^]^ as well as inhibiting bone fraction repair.^[^ ^35^ ^]^ Polymorphonuclear leukocyte extravasation through the vascular wall during inflammation depended on endothelial PIEZO1,^[^ ^36^ ^]^ and leukocyte PIEZO1 was found to promote bactericidal functions.^[^ ^37^ ^]^ Neutrophil PIEZO1 promoted pulmonary capillary angiogenesis.^[^ ^38^ ^]^ PIEZO1 may also have a role in fibroblast activation that promotes angiogenesis in tissue scarring.^[^ ^39^ ^]^ The cortex of lymph nodes where T cells encounter antigens includes the high endothelial venule supported by fibroblastic reticular cells.^[^ ^40^ ^]^ In mice, *Piezo1* disruption in the fibroblastic cells reduced their association with high endothelial venules, decreasing lymphocyte recruitment and antigen response.^[^ ^41^ ^]^ The pathophysiology section of this article lists many PIEZO contributions in disease and disease‐model experiments. Some of them may also be relevant in physiological responses to stress and injury and the associated regulations of vascular permeability.

### 3.7 Vascular Aging

Aging in mice was found to deplete PIEZO1 dependent peri‐arteriolar niches in load‐bearing bones and this was rescued partly by the mice running on a wheel, with accompanying increased bone thickness.^[^ ^28^ ^]^ Simulated microgravity to investigate the impact of space travel led to carotid artery aging‐like changes including increased stiffness, intima‐medial thickness, fibrosis, and elevation of senescence biomarkers in mice.^[^ ^42^ ^]^ *Piezo1* disruption in smooth muscle prevented these changes^[^ ^42^ ^]^ and inhibited aging‐induced circumferential stiffening of ascending thoracic aorta.^[^ ^43^ ^]^ Separate human genetic studies, discussed in the pathophysiology section of this article, suggest an important role of PIEZO1 in preserving lower limb venous integrity, which becomes particularly important in aging as shown by the common problem of varicose veins.

## 4 Channel Properties

To understand PIEZOs in physiology, their properties and molecular mechanisms need to be determined. Progress is outlined here for PIEZOs generally but with attention where possible to the vascular context because PIEZOs do not behave the same in all cells and situations. Understanding is most advanced for endothelial PIEZO1 and so this aspect is a focus.

### 4.1 Structure

High‐resolution microscopy and molecular dynamics simulations have provided important generic information for the structures and mechanisms of PIEZO channels.^[^ ^44^, ^45^, ^46^, ^47^, ^48^, ^49^, ^50^, ^51^, ^52^ ^]^ Three PIEZO1s or three PIEZO2s form PIEZO1 or PIEZO2 channels, each with 114 membrane‐spanning helices per channel and 38 per monomer. When the channels are closed and non‐conducting in the absence of mechanical stimuli, they strikingly dimple the membrane inwardly, creating an inverted dome with three N‐terminal blade‐like features extending out to the penumbra of the dome from the central C‐terminal ion pore region comprising the 37^th^ (outer) and 38^th^ (inner) helices of each monomer. The dimpling effect and blades are special features of PIEZO channels and seem to be critical in their unique force sensing properties. Linking and over the outer and inner helices is a large domain‐swapping C‐terminal extracellular domain (CED) that is somewhat like a bottle cap with feet touching down on nearby blade regions.^[^ ^46^ ^]^ Membrane stretch causes the dome to flatten and the blades to spread out radially, expanding the channel and triggering opening of the ion pore.^[^ ^49^, ^52^ ^]^ Inter‐blade monomer to monomer handshaking regulates the expansion depending on the membrane lipid phosphatidylinositol 4,5‐bisphosphate.^[^ ^52^ ^]^ A deeper dome and more embedded CED may restrict PIEZO2's mechanical sensitivity compared with PIEZO1.^[^ ^44^, ^48^ ^]^ Through these various mechanisms, the channel is thought to achieve regulated force sensing and downstream electrical signalling.

### 4.2 Activation

**Figure 1:**
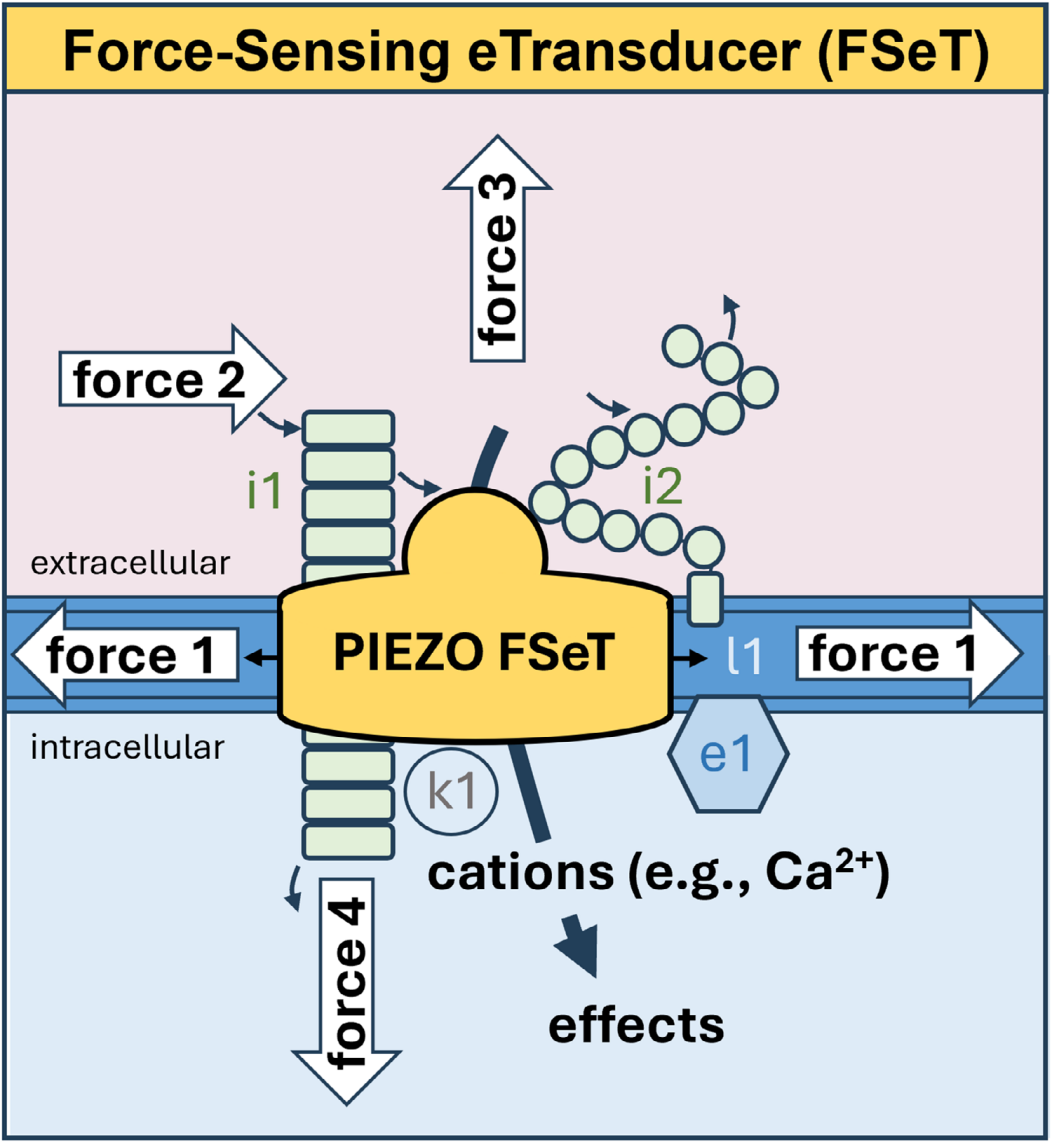
PIEZO channel Force‐Sensing eTransducer (FSeT) concept. Depicted is a section of cell membrane with membrane proteins that include a PIEZO channel (PIEZO FSeT), two PIEZO interacting proteins (i1 and i2; e.g., CADM1 and GPC1), a modulating membrane lipid (l1; e.g., ceramide) and associated proteins that include a kinase (k1; e.g., protein kinase A) and a lipid‐regulatory enzyme (e1; e.g., SMPD3). The semicircular structure depicted at the top of the PIEZO channel is the CED (i.e., cap structure). Four types of mechanical stimulus (forces 1‐4) are indicated: force 1 from membrane tension acting through lipids and other membrane components; force 2 from shear stress from fluid flow (e.g., blood flow or interstitial fluid flow) in extracellular compartments that include the blood vessel lumen, cell‐cell junctional compartments and interstitial compartments^[^ ^113^ ^]^; force 3 from tension in extracellular structures that include the glycocalyx and the extracellular matrices of intercellular compartments; and force 4 from tension in intracellular structures that include the cytoskeleton. Forces act directly on the PIEZO or on PIEZO via intermediate associated factors. In all cases, the PIEZO channel is the pivotal force sensor, serving to sense force and transduce it almost instantaneously into effects through pre‐existing potential energy from ionic gradients across the cell membrane (e.g., the large gradient for Ca^2+^ from the outside to the inside of the cell). Effects arising from the influx of Ca^2+^ are considered in Figure 2.

Electrical activities of native PIEZO channels have been relatively easily delineated because of the availability of patch‐clamp techniques with their high sensitivity, versatility and temporal resolution, as first shown for PIEZO1 in neuroblastoma cells^[^ ^1^ ^]^ and then endothelial and vascular smooth muscle cells^[^ ^2^, ^11^, ^17^, ^23^, ^53^, ^54^ ^]^ and other cell types.^[^ ^44^ ^]^ Patch‐clamp revealed that PIEZO channels are calcium ion (Ca^2+^)‐permeable non‐selective cationic channels of the plasma membrane, activating in response to mechanical forces from membrane stretch, cell poking by a probe, physiological fluid flow (shear stress) and substrate stiffness^[^ ^1^, ^2^, ^3^, ^44^, ^55^, ^56^ ^]^ (**Figure** **1**). Multiple modes of mechanical activation have been indicated that may serve PIEZO tuning to specific stimuli such as fluid flow and cytoskeletal tension^[^ ^57^, ^58^ ^]^ and enable PIEZO channels to sense and integrate multiple mechanical forces (Figure 1). The intrinsic force sensing abilities of these membrane‐embedded channels is complemented by additional factors such as associated proteins, as outlined in Figure 1 and evidenced in the sections below.

### 4.3 Sustained Activity

The initial patch‐clamp studies of neuroblastoma cells revealed rapid PIEZO channel activation and then almost as fast inactivation despite the mechanical stimulus being sustained.^[^ ^1^ ^]^ The inactivation, a switching off adaptive desensitization to continuous force, is also commonly seen in PIEZO channels experimentally overexpressed in HEK293 cells, which have been almost exclusively used by experimentalists as a host cell. This overexpression strategy has enabled studies of artificially mutated PIEZOs that have provided compelling evidence for inactivation being due to inherent properties of the PIEZO CED and central helices.^[^ ^59^, ^60^, ^61^ ^]^ It was surprising, therefore, to find that native PIEZO1 channels of endothelial and vascular smooth muscle cells did not inactivate or inactivated only slowly or incompletely.^[^ ^2^, ^23^, ^26^, ^53^, ^54^, ^62^, ^63^ ^]^ Native endothelial PIEZO1 channels of mouse mesenteric arteries were found, however, to inactivate if sphingomyelin phosphodiesterase 3 (SMPD3) was first inhibited pharmacologically or genetically, in which case the channel properties closely resembled those of overexpressed PIEZO1 channels with their fast and complete inactivation.^[^ ^53^ ^]^ Non‐inactivating behavior could be reinstituted in SMPD3‐inhibited endothelial cells by the addition of exogenous ceramide, suggesting that the SMPD3‐mediated catalysis of sphingomyelin to ceramide is what normally disables channel inactivation in endothelial cells, serving to promote sustained channel responses to force.^[^ ^53^ ^]^ An interaction of the SMPD3‐related SMPDL3B protein with PIEZO1 identified in an unbiased screen for protein partners^[^ ^64^ ^]^ has pointed to the possibility that sphingomyelinase activity may occur discretely with the channels to locally enrich ceramide for disabled inactivation (Figure 1).

### 4.4 Interacting Proteins

Some types of protein may interact directly to mediate or amplify effects of mechanical force and slow PIEZO1 channel inactivation to increase channel activity, such as the endothelium‐relevant proteins MDFIC (a transcriptional regulator), GPC1 and GPC4 (glypicans) and the CDH5 (VE‐cadherin), CADM1 and CADM4 cell adhesion molecules^[^ ^64^, ^65^, ^66^, ^67^ ^]^ (Figure 1). Alternatively, interaction with other cell adhesion molecules such as PECAM1 may inhibit channel activity.^[^ ^68^ ^]^ MDFIC has been observed embedded in the channel structure^[^ ^65^ ^]^ whereas glypicans and cell adhesion molecules interact with the CED.^[^ ^64^, ^67^ ^]^ The membrane span of cell adhesion molecules enables them to also couple PIEZOs to cytoskeletal partner proteins, thereby physically linking extracellular and intracellular environments and regulating cell‐cell junctions.^[^ ^67^, ^68^ ^]^ CED interaction with a soluble extracellular matrix protein called COMP has been found to enhance endothelial PIEZO1 channel activity,^[^ ^67^ ^]^ suggesting that the CED is a target for multiple regulatory molecules. Therefore, PIEZO1 channels seem to be decorated with other proteins extracellularly, cytosolically and within to facilitate context dependent regulation and integration.

### 4.5 Regulation by Phosphorylation

In brain, lung and umbilical cord endothelial cells, the activation of adenylyl cyclase, protein kinase A or protein kinase C was found to slow channel inactivation, increasing mechanically activated ionic currents and fluid flow‐evoked intracellular Ca^2+^ elevations by phosphorylating a cytosolic serine residue that precedes the 29^th^ helix of PIEZO1^[^ ^26^ ^]^ (Figure 1). A PIEZO1 splice variant that retains the phosphorylation event but not a lateral‐plug‐gate of the channel was unable to respond, suggesting that the gate transduces the serine phosphorylation into enhanced mechanical activation.^[^ ^26^ ^]^ Physiological modulators such as adrenomedullin and adrenaline acting via G_s_ protein‐coupled receptors may promote endothelial mechanical sensitivity through such a phosphorylation mechanism.^[^ ^26^ ^]^

### 4.6 Other Regulation

Knowledge of the regulation of PIEZOs is not exhaustively addressed in this article but the regulation is apparently extensive, mediated by interactions with membrane proteins such as cystic fibrosis transmembrane conductance regulator (CFTR)^[^ ^69^ ^]^ and β‐amyloid peptides,^[^ ^70^, ^71^ ^]^ transcriptional control through inflammatory mediators such as tumor necrosis factor α and microRNAs such as MiR‐103a^[^ ^72^, ^73^ ^]^ as well as lipid effects involving the cholesterol binding stomatin‐like protein 3 and factors such as 7‐ketocholesterol, margaric acid and docosahexaenoic acid.^[^ ^74^, ^75^, ^76^ ^]^

## 5 Downstream Signalling

**Figure 2:**
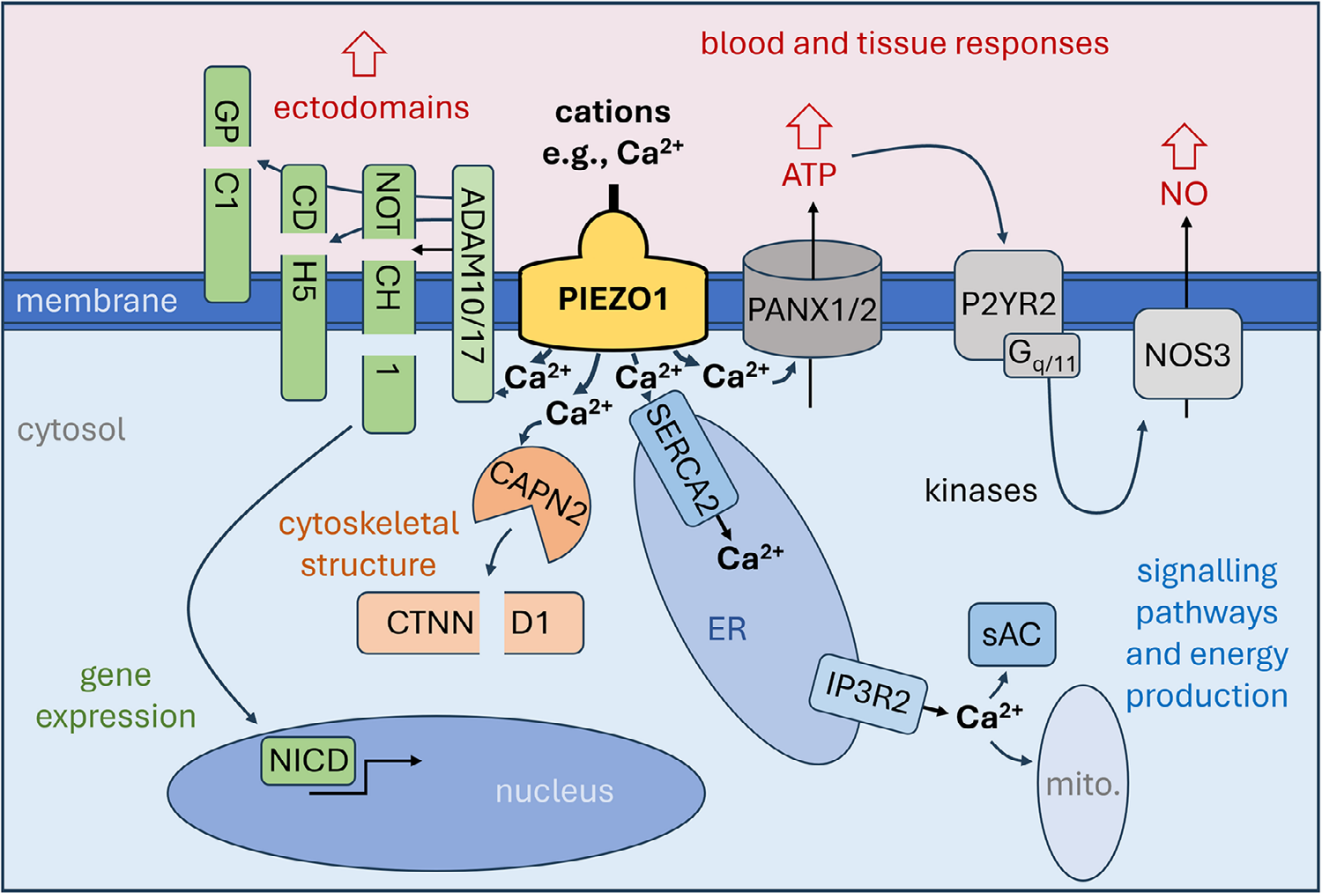
PIEZO1 downstream signalling concepts in a generic endothelial cell. Depicted is plasma membrane, cytosol and intracellular compartments. Intracellular compartments include nucleus, sarco‐endoplasmic reticulum (ER) and mitochondria (mito.). Extracellular compartments include glycocalyx and cell‐cell junctional structures. Other cells may be adjacent or otherwise nearby cells including other endothelial cells, abluminal mural cells such as pericytes and vascular smooth muscle cells, blood cells such as leukocytes that are luminal or migrating through the vascular wall, and parenchymal cells. The central focus of the diagram is the PIEZO1 channel, shown as a single shape in a mechanically activated state due to a force such as membrane tension or shear stress and thus with the ion pore open to allow ion permeation such as Ca^2+^ influx, driving a local rise in Ca^2+^ concentration at the intracellular face of the channel that triggers downstream events. As described and referenced in more detail in the main text, 4 types of downstream event are indicated following the rise in Ca^2+^ concentration: 1) Ca^2+^ activation of a calpain such as calpain‐2 (CAPN2) that cleaves downstream proteins such as p120‐catenin (CTNND1) for intracellular structural change via altered cytoskeleton; 2) Ca^2+^ activation of ADAM10 or ADAM17 to cleave downstream target proteins such as NOTCH1, CDH5 (VE‐cadherin) and glypican‐1 (GPC1) to release ectodomains for signalling to other cells and enable intracellular events such as γ‐secretase cleavage of NOTCH1 to release the NOTCH1 Intra Cellular Domain (NICD) for regulation of gene expression. 3) Ca^2+^ activation of pannexins 1 and 2 (PANX1/2) for efflux of ATP to act on nearby cells and feed back to the same cell via a G protein‐coupled receptor (P2YR2) and a G protein (G_q/11_) and thereby signal via a cascade of kinases to endothelial nitric oxide synthase (NOS3), leading to increased production of nitric oxide (NO) that diffuses to other cells to regulate their behavior; 4) Ca^2+^ uptake into ER via the SERCA2 pump, increasing the Ca^2+^ concentration inside the ER and promoting Ca^2+^ release via an inositol trisphosphate receptor‐channel (IP3R2) at a distant site in the cytosol to regulate factors such as soluble adenylyl cyclase (sAC) and the energy and free radical production of mitochondria (mito.). These downstream mechanisms enable the mechanical event to trigger: 1) intracellular restructuring; 2) inside‐out restructuring and outside‐in gene regulation; 3) paracrine signalling for coordination of cells nearby in the tissue and blood; and 4) coordination across the inside of the cell.

How PIEZO channel activation triggers downstream signalling for appropriate overall cell and tissue responses to mechanical force is a complex 4D problem that is far from being solved and highly likely to be cell type specific. Here, the focus is on a framework for the principles by which events occur downstream of PIEZO1 channel activation in endothelial cells where there is most substantial information (**Figure** **2**). What is shown and addressed below is a simplification that does not cover all available data or endothelial cell types in their different settings such as the lymphatics, arteries and capillaries, but it is hoped that the principles will have general value for understanding of what is involved and guide future studies in endothelial cells and potentially other cell types.

### 5.1 Beginning with Ca^2+^ Entry

When the channels open, there is influx of Ca^2+^ down the large pre‐existing electrochemical gradient for Ca^2+^ from the outside to the inside of the cell (Figure 2). Large elevations of the Ca^2+^ concentration in the immediate intracellular vicinity of the channels are likely to arise and be an important first step in the downstream signalling and ultimately the overall cell changes evoked by mechanical force. The channels also permit Na^+^ influx and K^+^ efflux (and possibly Cl^‐^ influx or efflux)^[^ ^77^ ^]^ but Ca^2+^ is special as a pivotal currency of intracellular control^[^ ^78^ ^]^ and trigger for plethora cell activities that include cell movement and secretion.^[^ ^79^ ^]^ Therefore, Ca^2+^ is emphasized. Ca^2+^ entry is not unique to these channels, however, as there are other Ca^2+^ permeable channels in the same cells, activated by other factors. Specific signalling may nevertheless be achieved for PIEZOs through special spatial positioning of the channels, PIEZO channel organization relative to other factors such as downstream signalling components and the details of the amplitudes, durations and frequencies of the PIEZO‐mediated Ca^2+^ events. Opposing cell extension and retraction phenomena have been seen for PIEZO1‐mediated Ca^2+^ entry depending on the intensity and frequency of the entry, showing that quantitative aspects of PIEZO channel activity can be critical.^[^ ^12^ ^]^

### 5.2 Calpain Activation for Intracellular Restructuring

Calpains are soluble cytosolic Ca^2+^ activated proteinases that cleave other intracellular proteins to modulate their characteristics.^[^ ^80^ ^]^ Unbiased proteomic studies first associated PIEZO1 with calpain‐2 and cytoskeletal substrates of calpains in endothelial cells.^[^ ^2^ ^]^ Stimulation of calpain activity by shear stress was prevented by a PIEZO1 inhibitor.^[^ ^2^ ^]^ Endothelial PIEZO1 deletion in mice strongly inhibited pressure‐evoked calpain‐dependent cleavage of p120‐catenin and Src kinase, which are key regulators of cytoskeleton and adherens junctions.^[^ ^81^, ^82^, ^83^ ^]^ PIEZO1‐dependent intracellular Ca^2+^ events of endothelial tip cells were unaffected by calpain inhibition while downstream tip retraction due to high frequency Ca^2+^ events was prevented,^[^ ^12^ ^]^ an association with high intensity events that is consistent with the activation of calpain‐2 requiring relatively high Ca^2+^ concentrations.^[^ ^80^ ^]^

### 5.3 ADAM Activation for Inside‐Out Restructuring and Outside‐In Gene Regulation

ADAM10 is a membrane‐spanning metalloproteinase activated by elevated cytosolic Ca^2+^ to cleave other membrane proteins near the extracellular membrane leaflet, thereby shedding ectodomains into the extracellular compartment.^[^ ^84^, ^85^ ^]^ It targets membrane proteins such as NOTCH1 and CDH5 that are key players in endothelial mechanical regulation.^[^ ^84^, ^86^, ^87^, ^88^ ^]^ Shear stress acting via PIEZO1 was found to stimulate ADAM10 and thereby drive downstream NOTCH1 activation by enabling γ‐secretase to release the NOTCH1 intracellular domain (NICD), driving expression of genes such as *HES1*.^[^ ^89^ ^]^ NOTCH1 activation of this type may be crucial in how blood flow acts on endothelial cells to regulate junctional integrity^[^ ^88^ ^]^ and stimulate pericyte recruitment.^[^ ^14^ ^]^ PIEZO1 may alternatively activate the ADAM10‐related ADAM17, cleaving TIE1 to drive lymphatic valve formation via FOXO1‐mediated gene regulation.^[^ ^90^, ^91^ ^]^ Both of these ADAMs cleave components of the endothelial glycocalyx,^[^ ^92^ ^]^ an extracellular structure containing glycoproteins and proteoglycans at the luminal surface of blood vessels.^[^ ^93^ ^]^ Physical interaction of ADAM10 and ADAM17 with PIEZO1^[^ ^64^ ^]^ may facilitate the specific activation of these proteinases by PIEZO1‐mediated Ca^2+^ entry.

### 5.4 Pannexin Activation for Paracrine Signalling

Pannexins are Ca^2+^ activated transmembrane channels that mediate the release of cytosolic ATP down its gradient to the extracellular compartment.^[^ ^94^, ^95^, ^96^ ^]^ There is evidence that PIEZO1 channels use the pannexin route in endothelial cells, thereby activating cell surface P2Y2 ATP receptors that trigger an intracellular kinase cascade, ultimately altering endothelial NO synthase (NOS3) phosphorylation to increase production of the omnipotent diffusible messenger, NO.^[^ ^24^, ^97^ ^]^ NOS3 is itself Ca^2+^ dependent,^[^ ^98^ ^]^ so might also be stimulated more directly by PIEZO1‐mediated Ca^2+^ entry. There is clearly complexity in PIEZO1‐driven NOS3 regulation^[^ ^99^ ^]^ with key details still to be elucidated by future research.

### 5.5 Ca^2+^ Store Loading for Intracellular Crosstalk

SERCA proteins of the intracellular sarco‐endoplasmic reticular (ER) membranes bind cytosolic Ca^2+^ to pump it into ER compartments for controlled Ca^2+^ release back to the cytosol via other proteins. PIEZO1 was found to interact with SERCA2, suggesting juxtaposed plasma membrane PIEZO1 channels and ER membrane SERCA2 pumps.^[^ ^62^ ^]^ In endothelial cells, PIEZO1 channels were found to elevate the Ca^2+^ concentration inside the ER through SERCA activity, promoting Ca^2+^ release via inositol 1,4,5‐trisphosphate receptor type 2 (IP3R2) to spread the Ca^2+^ signal to soluble adenylyl cyclase and potentially mitochondria.^[^ ^100^, ^101^, ^102^ ^]^ The involvement of intracellular Ca^2+^ stores may explain the participation in PIEZO1 effects of the downstream mediator ORAI1,^[^ ^103^ ^]^ which maintains the Ca^2+^ content of vascular ER through focused Ca^2+^ entry.^[^ ^104^ ^]^

### 5.6 Signalling Complexity

From the origins of these Ca^2+^‐triggered events (summarized above and in Figure 2) many more effects can be expected via other signalling pathways, gene regulation, secreted factors and cell processes as time progresses from the initial mechanical stimulus. Indeed many effects have been observed downstream of PIEZO1 activation in endothelial and other cell types such as vascular smooth muscle cells.^[^ ^6^, ^24^, ^29^, ^34^, ^54^, ^90^, ^99^, ^103^, ^105^, ^106^, ^107^, ^108^, ^109^, ^110^, ^111^ ^]^

## 6 Flow Sensing

Pivotal for the cardiovascular system is the endothelial sensing of shear stress from the flow of blood or lymph.^[^ ^93^, ^112^, ^113^, ^114^ ^]^ This sensing serves to match the architectures and properties of the vasculature and cardiac and vascular valves to the demands of the heart and other organs. PIEZO1 has key roles in many aspects of this capability as already indicated. The molecular mechanisms of the system are, however, not restricted to PIEZO1. They are instead rather complex, controversial and relatively poorly understood despite enormous research effort.^[^ ^115^ ^]^ A commonly described hypothesis is that PIEZO1 is one amongst many diverse molecules sensing force in parallel and interconnecting relatively vaguely.^[^ ^30^, ^112^, ^113^, ^115^, ^116^, ^117^, ^118^, ^119^ ^]^ The molecular site at which the force is detected and transduced is quite unclear and commentaries rather portray parallel multidetector arrangements with shear stress diffusely positioned above. While parallel sensors could guarantee force sensing in the event of any one detector failing, the studies that propose the sensors have often reached their conclusions precisely because the system failed if the candidate molecule was disrupted, thus arguing against multiple sensors providing backups or fail‐safes for each other. Here, the multidetector model is challenged with an Occam's razor for PIEZO, particularly PIEZO1, channels as the sole force sensors adapting to different endothelial settings and conferring force sensitivity on other components that are important in the system but not as force sensors. In this model, PIEZO channel is depicted as a Force‐Sensing eTransducer (FSeT), a kernel with better and more selective force sensing than other types of protein and a key ability to rapidly and efficiently transduce the detected force into cell and tissue effects through electrical (e) signalling (Figure 1).

### 6.1 Fast Direct Force Sensing

The discovery of PIEZOs provided a special opportunity for understanding fluid flow sensing because these proteins are ion channel subunits that can be studied by patch‐clamp, which is an exceptional high‐fidelity recording technique capable of millisecond time resolution of single or multiple ion channel activities and determination of responses to fluid flow in excised cell membrane patches that greatly reduce or remove complications from other cell components such as intracellular organelles.^[^ ^1^, ^23^, ^120^ ^]^ The technique has revealed impressive capabilities of PIEZO channels as force sensors. Overexpression of PIEZO1 in the host non‐endothelial HEK293 cell was found to confer ionic currents that were fluid flow‐activated within milliseconds.^[^ ^3^ ^]^ Native PIEZO1 channels of endothelial cells were similarly found to be activated rapidly by fluid flow in cells and excised outside‐out patches.^[^ ^2^, ^17^, ^23^, ^53^ ^]^ A robotic planar patch‐clamp system showed rapid activation of PIEZO1 channels by fluid ejected from a pipette.^[^ ^121^ ^]^ The idea that PIEZO1 channels are rapidly activated by fluid flow has also been supported by studies using independent techniques. Because the channels are Ca^2+^ permeable and Ca^2+^ enters cells via the channels to raise cytosolic Ca^2+^ quickly, PIEZO activity can be separately measured by a chemical or genetically encoded cytosolic Ca^2+^ indicator, or a genetically encoded indicator that senses conformational change in the channel. Such indicators are relatively easily deployed and less invasive to cells than patch‐clamp. Importantly, studies with indicators of this type have similarly suggested rapid activation of overexpressed and native endothelial PIEZO1 channels by fluid flow.^[^ ^2^, ^26^, ^70^, ^122^, ^123^, ^124^, ^125^ ^]^ In separate studies, fluid flow was found to alter membrane lipids, even in artificial lipid bilayers without proteins, but the effects were slow in time course relative to the activation of PIEZO1 channels and so seem not to explain the rapid activation of PIEZO1 channels.^[^ ^126^, ^127^, ^128^, ^129^ ^]^ Therefore, although PIEZO channels are integrated with lipids, the channels are suggested as the molecular machines that sense force and transduce force into effects (Figure 1).

An alternative and more commonly used experimental mechanical stimulus than fluid flow is membrane tension achieved through pressure applied to the inside of the patch pipette, stretching the membrane at the tip of the pipette to mechanically alter the environment of the channels.^[^ ^55^ ^]^ Overexpressed and native endothelial PIEZO1 channels are activated by this stimulus type.^[^ ^2^, ^26^, ^53^ ^]^ While the channels may respond to fluid flow indirectly via changes in membrane tension (Figure 1), artificial PIEZO1 proteins incorporating cyclic permuted green fluorescent protein have been generated that give fluorescent readouts specific to fluid flow and not membrane tension, suggesting PIEZO1 conformations peculiar to the sensing of fluid flow.^[^ ^57^ ^]^ The relevance of dome flattening and radial blade spreading to the fluid flow‐specific effects remain to be determined. Fluid flow, membrane stretch and other forces such as stiffness may all be important in the physiological setting of the channels, interacting with each other (Figure 1).

There is, therefore, strong evidence that PIEZO1 channels are activated by fluid flow, responding rapidly and apparently without the need for intermediates. They are, nevertheless, membrane protein complexes that depend on their integration with membrane lipids and associated other proteins that may help convey force to the channels in native environments. The channels respond to fluid flow whether overexpressed in a host cell that is intrinsically flow resistant (i.e., HEK293 cells) or in endothelial cells that are intrinsically flow sensitive. Evidence of this type is mostly not available for other candidate sensors of the endothelial flow sensing machinery, which are often more challenging to study in short timeframes and have been proposed as sensors based on relatively slow stimuli and readouts.^[^ ^122^, ^130^, ^131^, ^132^ ^]^ It cannot be excluded that other candidate sensors might also exhibit rapid responses if they could be studied in similar ways to PIEZO channels, but in several cases other candidates have also been ion channels studied by patch‐clamp and none has shown the robust, rapid and apparently direct sensitivity to fluid flow of PIEZO1 channels.^[^ ^16^, ^115^, ^133^ ^]^ Some force sensors may simply respond relatively slowly and still be relevant in the time scales of endothelial physiology but the existence of a robust fast‐responding sensor (i.e., PIEZO channel) in the same cells creates a plausibility that slow responses are consequences of earlier PIEZO activation.

### 6.2 Integration with other Candidate Sensors

PIEZO1 channels meet criteria to be a force sensor more readily than many candidate sensors^[^ ^115^ ^]^ but also, unusually for candidate sensors, they are increasingly identified as partners of many of the other candidate sensors, thereby offering potential explanations for how other factors have been considered as sensors or modulators of sensing. Candidates found to be downstream or otherwise associated with PIEZO1 include the cell adhesion molecules PECAM1 and CDH5,^[^ ^24^, ^68^, ^87^ ^]^ the glycocalyx glypican‐1 (GPC1),^[^ ^64^, ^93^, ^131^ ^]^ AKT kinase and NOS3,^[^ ^2^, ^24^, ^134^ ^]^ SMPD3 (neutral sphingomyelinase) and ceramide,^[^ ^53^, ^135^, ^136^ ^]^ sphingosine‐1‐phosphate,^[^ ^137^ ^]^ NOTCH1 and NOTCH signalling,^[^ ^86^, ^89^, ^103^ ^]^ integrins,^[^ ^122^, ^138^, ^139^ ^]^ Src kinase and the transcriptional coactivator YAP1,^[^ ^110^, ^140^, ^141^, ^142^ ^]^ TRPV4 channel,^[^ ^124^, ^133^, ^143^ ^]^ polycystin‐2 channel^[^ ^144^, ^145^ ^]^ and ATP‐release, P2Y2 receptor and the Gα_q/11_ protein.^[^ ^24^ ^]^ Like the P2Y2 receptor, the P2X4 receptor may have been implicated in flow sensing^[^ ^146^ ^]^ because PIEZO1 drives ATP release.^[^ ^24^ ^]^

### 6.3 Luminal Barrier

Force from the flow of blood or lymph is likely to be intimately experienced by glycocalyx, a luminal extracellular structure that may interfere with the impact of shear stress on PIEZO1 channels and other integral membrane proteins.^[^ ^93^, ^147^ ^]^ While the glycocalyx might partly or fully shield the channels, it may also transmit force to the channels through associated glypicans of the glycocalyx^[^ ^64^, ^93^ ^]^ and through membrane tension from flow‐mediated mechanical disturbance of membrane‐anchored glycoproteins (Figure 1).

### 6.4 Sensor for all Flows

Shear stress at the endothelium varies greatly due to differences in the intensity, uniformity and patterns of blood and lymph flow depending on vascular anatomy and parameters such as blood pressure.^[^ ^113^, ^148^ ^]^ PIEZO1 channels seem adaptable to these variations, serving as general sensors of laminar and oscillating shear stresses, even though the resulting downstream signalling may differ strongly.^[^ ^139^ ^]^ There is partial insight for how a different signalling paradigm may arise from the same sensor (i.e., PIEZO1)^[^ ^139^ ^]^ but much remains to be understood.

### 6.5 PIEZO‐Centric Thinking

A model should therefore be considered in which PIEZO channels are the pivotal force sensors of the endothelial flow sensing machinery, directly sensing force from flow, sensing force transmitted from flow via other proteins or the membrane and transducing force rapidly into effects (Figures 1 and 2). While PIEZO1 is dominant, it may on occasions be replaced or joined by PIEZO2,^[^ ^11^, ^149^ ^]^ hence the suggestion of a PIEZO‐ rather than PIEZO1‐centric hypothesis. The PIEZO channels are referred to as Force‐Sensing eTransducers (FSeT) (Figure 1) to indicate their dual function as sensor and electrical (e) transducer, tapping into the powerful ionic gradients of cells to rapidly and efficiently convert force into effect via the flux of Ca^2+^ and other ions.

## 7 Pathophysiology and Therapeutics

In addition to physiological roles, PIEZOs importantly participate in vascular pathophysiology, apparently via two types of mechanism. One is through gene, primarily *PIEZO1* gene, variation that substantially alters the channel expression or function. Consistent with the suggested importance of PIEZOs in vascular physiology, adverse vascular effects dominate the disease outcomes of disruptive variants, as outlined below and presented in more detail elsewhere.^[^ ^150^ ^]^ The second mechanism seems to be independent of the gene variants but rather through PIEZOs responding adversely to altered force in a tissue, perhaps because of injury to the tissue or aging processes. Mechanical stresses of this type bring vascular smooth muscle PIEZO1 into play.^[^ ^54^ ^]^

### 7.1 Genetic Association 1: Generalized Lymphatic Dysplasia

Generalized lymphatic dysplasia (GLD) is a rare primary lymphedema due to lymphatic abnormality.^[^ ^151^ ^]^ Studies of families affected by a specific type of GLD called Fotiou GLD and characterized by a high incidence of NIFH and fetal demise,^[^ ^8^, ^151^, ^152^ ^]^ have suggested *PIEZO1* variants as a common underlying cause due to the ability of the variants to disrupt PIEZO1 protein expression, localization or function.^[^ ^8^, ^150^, ^153^, ^154^, ^155^ ^]^ The disease has been associated particularly with variants that are homozygous or compound heterozygous such that the disease is mostly absent if there is a single heterozygous variant, with parents of the GLD suffers often not presenting with the disease themselves.^[^ ^8^, ^153^, ^154^, ^155^ ^]^ In cases where some PIEZO1 is still available but reduced or less functional, targeting this residual PIEZO1 in a potential therapeutic strategy may be considered.^[^ ^154^, ^155^ ^]^

### 7.2 Genetic Association 2: Varicose Veins

Varicose veins are common and, although often benign, frequently a burden to health services and individuals. They are a manifestation of chronic lower limb venous hypertension that leads to venous valve incompetence and distorted superficial veins particularly in older people.^[^ ^156^ ^]^ Associated chronic venous congestion and loss of valve competence may increase the risk of deep vein thrombosis with genetic components of the disease occurring alongside environmental and lifestyle factors.^[^ ^156^, ^157^ ^]^ Unbiased genome‐wide investigations have strongly associated *PIEZO1* variants with this disease.^[^ ^158^, ^159^, ^160^, ^161^ ^]^ These findings suggest that PIEZO1 is important in the maintenance of lower limb venous integrity with aging and raise the possibility that targeting PIEZO1 could be useful as an intervention.

### 7.3 Genetic Associations with other Vascular and Vascular Related Problems

There are clinical features of some Fotiou GLD patients and other people harboring *PIEZO1* variants that may be a consequence of lymphatic disruption or alternatively reflect other vascular roles of PIEZO1. These other features include maternal and fetal vascular malformations, venous valve anomaly, double superior vena cava, agenesis of the ductus venosus, bilateral periorbital and conjunctival vascular changes with small punctate hemorrhages and deep vein thrombosis.^[^ ^150^ ^]^ People who apparently do not suffer from lymphedema have associations of *PIEZO1* variants with other vascular and vascular related phenomena such as cerebral cavernous and brain arteriovenous malformations and bicuspid aortic valves.^[^ ^5^, ^150^, ^162^, ^163^ ^]^ Loss of function effects of *PIEZO1* variants may be the underlying cause but experimental evidence is awaited to demonstrate this in many cases.^[^ ^150^ ^]^ Contrasting gain of function *PIEZO1* variants have been associated not with vascular disease but rather with red blood cell disorders that include a dehydrated hereditary stomatocytosis.^[^ ^150^ ^]^ This disturbance of red blood cells has implications for interpreting the clinical biomarker HbA1c,^[^ ^164^ ^]^ which is used to diagnose diabetes, a common comorbidity of cardiovascular disease. There is less information linking *PIEZO2* variants with vascular disease but there are suggested associations with abnormal coronary vascular development^[^ ^11^ ^]^ and endomysial capillary microangiopathy.^[^ ^165^ ^]^

### 7.4 Non‐Genetic Associations

There is rapidly expanding evidence for PIEZOs contributing importantly to many other vascular and vascular related disease problems regardless of genetic associations (**Tables** **1** and **2**). The evidence has arisen from studies of preclinical animal models of human diseases as well as patient‐derived cells and tissues. Studies of *PIEZO* genetics have not associated PIEZOs with these conditions either because *PIEZO* variants are not relevant or because the diverse, multifactorial, life‐course, environmental and polygenic characteristics of these conditions preclude the detection of variant contributions. PIEZOs might instead participate in these diseases through changes in their abundance, posttranslational modification or activity, possibly alongside confounding alterations in other factors. There are risks that preclinical animal models of disease give misleading impressions of human disease but, even if this is the case, the results provide compelling evidence that PIEZOs can contribute importantly and adversely when tissue is damaged and thus altered in structure and mechanical properties.

**Table 1:** Vascular and vascular related problems that might be addressed by a PIEZO enhancer. Listed in the table are disease problems that may arise from or be exacerbated by abnormally low PIEZO expression or activity. The problems may be addressed by a PIEZO enhancer (e.g., PIEZO agonist) that improves the expression or function of the remaining available PIEZO channels. The conditions mostly involve PIEZO1 channels, but individual studies should be consulted for details of the PIEZO type.

Lymphedema, craniosynotosis, hydrocephalus, Down syndrome^[^ ^8^, ^90^, ^103^, ^153^, ^154^, ^155^, ^169^, ^170^, ^171^, ^172^ ^]^
Fetal growth restriction,^[^ ^173^ ^]^ uterine flow restriction,^[^ ^174^, ^175^ ^]^ early‐onset preeclampsia^[^ ^176^ ^]^
Erectile dysfunction^[^ ^175^ ^]^
Dysregulated cardiac metabolism^[^ ^107^ ^]^
Aortic aneurysm in Marfan Syndrome^[^ ^177^ ^]^
Thoracic aortic aneurysm^[^ ^178^ ^]^
Atherosclerosis^[^ ^179^ ^]^
Systolic hypertension,^[^ ^24^, ^180^ ^]^ baroreflex regulating arterial blood pressure^[^ ^181^ ^]^
Pulmonary hypertension^[^ ^149^, ^182^, ^183^ ^]^
Ventilator‐induced lung injury^[^ ^81^ ^]^
Intestinal ischemia‐reperfusion injury^[^ ^182^ ^]^
Ulcerative colitis and sepsis^[^ ^184^ ^]^

**Table 2:** Vascular and vascular related problems that might be addressed by a PIEZO inhibitor. Listed in the table are disease problems that may arise from or be exacerbated by abnormally high PIEZO expression or activity, and which may, therefore, be addressed by a PIEZO inhibitor (e.g., PIEZO antagonist) that tempers the excessive PIEZO channels. The conditions mostly involve PIEZO1 channels, but individual studies should be consulted for details of the PIEZO type.

Varicose vein disease^[^ ^185^ ^]^
Arteriovenous malformations^[^ ^186^ ^]^
Abdominal aortic aneurysm, thoracic aortic aneurysm^[^ ^187^, ^188^ ^]^
Aortic valve stenosis, calcific aortic valve disease^[^ ^189^, ^190^ ^]^
Atherosclerosis^[^ ^75^, ^109^, ^110^, ^139^, ^140^, ^141^, ^191^, ^192^, ^193^, ^194^, ^195^, ^196^, ^197^, ^198^ ^]^
Arterial calcification^[^ ^199^, ^200^ ^]^
Hyperlipidemia^[^ ^29^ ^]^
Systemic hypertension^[^ ^54^, ^201^, ^202^, ^203^ ^]^ with renal damage^[^ ^204^, ^205^ ^]^
Neointimal hyperplasia^[^ ^206^, ^207^ ^]^
Pulmonary hypertension^[^ ^63^, ^149^, ^208^, ^209^, ^210^ ^]^
Pulmonary edema from aortic stenosis^[^ ^81^ ^]^
Ventilator‐induced lung injury^[^ ^211^ ^]^
Pulmonary vascular damage from SARS‐CoV‐2 infection^[^ ^212^ ^]^
Radiation‐induced lung injury^[^ ^213^ ^]^
Portal hypertension^[^ ^214^, ^215^ ^]^
Hepatobiliary injury^[^ ^216^, ^217^ ^]^
Acute kidney injury and endothelial dysfunction in hyperglycemia^[^ ^218^, ^219^ ^]^
Lower limb ischemia, peripheral arterial/ vascular disease^[^ ^220^ ^]^
Intracerebral hemorrhage^[^ ^221^, ^222^ ^]^ and cerebral ischemia‐reperfusion injury^[^ ^223^ ^]^
Vibration‐induced vascular injury^[^ ^224^ ^]^
Scar tissue keloids^[^ ^39^, ^225^ ^]^
Tortuous vascularity of psoriasis^[^ ^226^ ^]^
Neuropathic vascular motion induced pain,^[^ ^227^ ^]^ diabetic peripheral neuropathy^[^ ^228^ ^]^
Vascular aging^[^ ^42^, ^43^, ^229^, ^230^ ^]^
Vascular dementia and disrupted blood brain barrier^[^ ^231^ ^]^
Tumor/ cancer angiogenesis, metastatic extravasation^[^ ^232^, ^233^, ^234^, ^235^ ^]^

### 7.5 Potential for New Therapeutics

Relevance of PIEZOs to unsolved vascular diseases (Tables 1 and 2) is a motivation to develop strategies for artificially modulating PIEZOs, for example through pharmacological approaches. There is already early‐stage progress with PIEZO pharmacology, particularly for PIEZO1.^[^ ^166^ ^]^ A commonly used small molecule positive enhancer (agonist) is Yoda1, which activates PIEZO1 but not PIEZO2 channels.^[^ ^166^ ^]^ A Yoda1‐related small molecule, Dooku1, inhibits PIEZO1 channels, and the spider toxin, GsMTx4, inhibits PIEZO1 and PIEZO2 without being selective for either.^[^ ^166^ ^]^ The suggested vascular and vascular related diseases that could potentially be addressed by a PIEZO enhancer most commonly involve PIEZO1, as in lymphedema for example (Table 1). There are a few suggested uses for PIEZO2 enhancers but here a PIEZO2 agonist strategy would need to be devised because PIEZO2 channel agonists are not yet reported.^[^ ^166^ ^]^ PIEZOs could alternatively be modulated indirectly or through genetic manipulation but, regardless of the strategy, much more progress is likely to be necessary if PIEZO‐targeted therapeutics are to be achieved.

While there is progress and interest in PIEZO modulation, there are major challenges to the idea of targeting PIEZOs because of seemingly opposing roles of the same PIEZO in apparently similar disease (Tables 1 and 2). While beneficial effects of a PIEZO1 agonist in atherosclerosis have been suggested, there are many more suggestions of potential benefits of PIEZO1 inhibitors in this condition (Tables 1 and 2). Aneurysms, hypertension and ventilator‐induced lung injury have also been suggested to be addressed by agonists or antagonists (Tables 1 and 2). Complexities in the types and mechanisms of these diseases need further investigation before a PIEZO modulation strategy can be devised. Different results may arise for many reasons such as the time‐dependent natures of disease progression. It is also necessary to consider PIEZO roles in non‐vascular cell types such as monocytes and contextual factors such as the amount of whole‐body physical activity and the age of the intended target individuals.

Potentially beneficial effects of PIEZO modulation have been observed particularly with the PIEZO1 agonist Yoda1, as reviewed elsewhere.^[^ ^166^ ^]^ However, adverse effects of PIEZO modulation might also be anticipated because of the developmental and adult physiology roles of PIEZOs and other roles of PIEZOs not covered here including roles in immunity^[^ ^167^ ^]^ and cardiac arrhythmia.^[^ ^168^ ^]^ Nevertheless, PIEZO1 activators and non‐specific PIEZO inhibitors have been administered to animals, leading to apparently beneficial effects.^[^ ^166^ ^]^ Therefore, adverse effects might be manageable if there is efficacy against a disease that is life‐threatening or strongly reduces a person's quality of life.

## 8 Forward‐Looking Perspectives

Despite the tremendous new knowledge, it is almost certainly only a glimpse into the true scope and depth of this biology. As ever, discoveries and conceptual challenges bring new questions. Future research will likely generate more knowledge and opportunities and refine and change concepts, much of which is difficult to predict but, here, a few forward‐looking perspectives are suggested to inform the debate about what to prioritize next.

### 8.1 Developmental and Adult Physiology

The importance of endothelial PIEZO1 in vascular physiology stands out in the results obtained so far but PIEZO1 in other cell types such as vascular smooth muscle might contribute more than is currently recognized. Genetic disruption of smooth muscle PIEZO1 has lacked the dramatic vascular physiology effects^[^ ^54^ ^]^ seen with endothelial disruption^[^ ^2^, ^3^ ^]^ but smooth muscle PIEZO1 has been found to be important when stress is applied experimentally to model vascular disease (Table 2). Vascular smooth muscle PIEZO1 seems unlikely to exist to cause disease and so may have importance in physiology not yet appreciated, perhaps becoming apparent if the limited physical challenges normally experienced by laboratory animals are increased to those of wild environments. If more physically vibrant and challenging conditions are created, do the roles of the PIEZO force sensors change; for example, are whole body physical activities such as voluntary running relevant to the roles of vascular smooth muscle PIEZO1, as they are for endothelial PIEZO1? The vasculature also comprises adventitial and other perivascular fibroblasts influencing vessel integrity and stiffness. Roles of PIEZO1 occur in cardiac fibroblasts,^[^ ^236^, ^237^, ^238^ ^]^ so PIEZO1 may be similarly significant in vascular fibroblasts, depending on physical activity.

Another prominent outcome of the research so far is the importance of PIEZO1 relative to PIEZO2, but is there more to appreciate about PIEZO2? Are there vascular neuronal functions of PIEZO2 not yet known, as in reflexes beyond those of the baroreceptors? Is perivascular neuronal PIEZO2 a general driver of vascular development across diverse organs? Endothelial PIEZO2 contributes to coronary development but is this peculiar to the heart and, if so, why? How does the extremely short‐lived activity of PIEZO2 channels^[^ ^11^ ^]^ contribute to the relatively slow behaviors of coronary endothelial cells that apparently do not involve rapid electrical firing patterns of neuronal physiology? Why is PIEZO2 selected for developing but not mature coronary vasculature?

Most animal research uses young animals but the roles of PIEZOs are likely to change in old age, for example because there is reduced physical activity of old animals, life course injury responses modify tissue structures and vascular stiffness increases. Research on old animals is challenging but there is potential importance of it here because of the prevalence of old age in human populations, old age being a major driver of cardiovascular disease and the possibility for new fundamental understanding of aging processes such as the contributions and molecular foundations of shifting mechanical set points.

### 8.2 Channel Properties, Structure and Partners

Many questions remain about how PIEZO1 and PIEZO2 channels work in molecular detail and tune their force sensitivities to specific cell needs. Protein structural knowledge is important for answering such questions but it is still relatively primitive and incomplete for example in the proximal N‐terminal blade regions of the channels, possibly because these regions have high physical mobility that blurs their microscopic resolution.^[^ ^52^, ^239^ ^]^ Although there is sufficient structural resolution in parts of the channel to predict molecular mechanisms, the clarity is mostly inadequate for understanding key processes or the details of associations with modulatory molecules such as lipids. The structural data are mostly for static channel states and the channels reconstituted in detergent or a simple non‐physiological lipid environment, yet the natural channels are almost certainly highly dynamic and integrated with complex lipids and other proteins (Figure 1). Some limitations have been overcome by advanced microscopy^[^ ^240^, ^241^ ^]^ and computer‐based molecular dynamics simulations in endothelial membrane,^[^ ^52^ ^]^ the continued application of which should lead to further new advances.

There are still relatively few molecular details for native PIEZO channels beyond their predicted amino acid sequences and, in some cases, posttranslational modifications such as the presence or absence of N‐linked glycosylation. As indicated already, PIEZOs do not behave or signal the same in all cells. Context‐specific understanding is most advanced for endothelial PIEZO1 but even here the knowledge is insufficient for full understanding, and for other cell types such as vascular smooth muscle cells and neurons and for PIEZO2 there is almost no cell type‐specific knowledge. Refinement of existing laboratory techniques and new methods may be essential to visualize details of the channels in native physiological environments. The new methods are likely to include cryo‐electron tomography, but major advances are needed in such methods if the native channels are to be visualized experimentally in natural cell and tissue environments with sufficient molecular detail for understanding of PIEZO channels as integrated dynamic force sensors. It may be that, as in other areas of research, computational approaches become essential, generating detailed molecular hypotheses for iterative refinement by laboratory testing.^[^ ^52^ ^]^ These approaches may be especially important in efforts to relate molecular details of the channels to key properties such as regulated inactivation and ion conduction in proportion to specific forces. Computational approaches may also inform understanding of the differences between PIEZO1 and PIEZO2 channels and the contributions of their associated proteins and lipids to different modes of their activation in different cell types.

Protein partners and modulators of endothelial PIEZO1 have been identified but there is still much to know and understand. The genetic disruption of SMPD3 profoundly converted native endothelial PIEZO1 channels to fast inactivating channels, suggesting SMPD3's sufficiency in explaining the normal slow or non‐inactivating property of these PIEZO1 channels.^[^ ^53^ ^]^ However, there are other regulators to consider. The MDFIC partner protein was identified in other cell systems^[^ ^65^ ^]^ and warrants investigation in the vascular context. Does it also regulate endothelial PIEZO1 inactivation and, if so, how do its effects relate to those of SMPD3? Are these protein partners relevant to other vascular PIEZOs such as vascular smooth muscle PIEZO1 and baroreceptor PIEZO2? Does lipid modulation of PIEZOs enable crosstalk with other molecular systems such as G protein‐coupled receptors that potentially deplete phosphatidylinositol 4,5‐bisphosphate at PIEZO channels to enhance their force sensitivity^[^ ^52^ ^]^?

If new knowledge such as this can be obtained, it may be possible to explain how PIEZOs satisfy the different needs of different cell types and contexts across vascular systems and associated systems such as the immune system. It may help understanding of why PIEZO2 is selected in some vascular biology over PIEZO1. Alongside this research, it will be helpful to determine the mechanisms controlling the quantity, localization and posttranslational modifications of PIEZOs because these mechanisms may prominently determine PIEZO contributions.

### 8.3 Downstream Signalling

How does the full complexity of PIEZO downstream signalling work and depend on the type of mechanical stimulus and cell type? A major challenge for researchers is understanding the organizations, timings and cell type‐specific effects of signalling downstream of PIEZOs. In future studies, particular attention should be paid to signalling activated by physiological forces in physiological settings and the time‐dependent sequences of short‐ and long‐term events as well as the reestablishment of stasis once a new mechanical context stabilizes. It will be important to understand signalling in different cell types, and for the two PIEZO types and other PIEZO channel diversities arising from microenvironments of their interacting proteins, lipids and posttranslational modifications (Figure 1). There is much to consider. If the channel is activated mechanically, do effects originate only from the PIEZO channel? Are there synergistic or competing mechanical pathways? How do spatial factors contribute, such as the directionality and history of the force application^[^ ^139^ ^]^ and the subcellular compartmentalization of PIEZOs^[^ ^68^, ^194^, ^208^, ^242^ ^]^? Spatial factors are likely to be critical in situations such as the orientation of endothelial cells in the direction of fluid flow. Membranes resist transmitting mechanical stimuli beyond the local perturbation^[^ ^243^ ^]^ and so localized subcellular factors are likely to be important in how PIEZOs are controlled. If the PIEZO channel is activated by a pharmacological agonist for better specificity than mechanical force, are artificial effects seen, for example, from sustained global rises in the intracellular Ca^2+^ concentration that lack physiological correlates? Could controlling the agonist in concentration, space and time provide better specificity and mimicry of the physiological force situation? Subcellular localizations, amplitudes and rates of rise and fall of PIEZO‐mediated Ca^2+^ changes seem likely to be key determinants of the ultimate outcomes of the effects of mechanical force on the cells. There is much still to learn about this.

Better understanding of PIEZO1‐driven NOS3 regulation will be particularly important because of its implications for understanding the mechanisms of flow‐mediated endothelium‐dependent vasodilatation, endothelial cell properties such as tip cell extension, protection of endothelial cells against apoptosis and other effects such as the control of gene expression in nearby parenchymal cells.^[^ ^12^, ^24^, ^25^, ^29^ ^]^

### 8.4 Flow Sensing

A controversial concept is the idea of PIEZOs as special force sensors in endothelial flow sensing, conferring force sensitivity on other proteins and acting as the primary force sensor rather than a force sensor amongst equals. This concept challenges prevailing thinking on how major elements of cardiovascular biology arise in response to fluid flow and how they adapt or maladapt, for example in atherosclerosis. Further testing of this concept will be important in future research. Is a PIEZO‐centric hypothesis for endothelial flow sensing, correct? PIEZO channels are integral membrane proteins that depend on their relationships with lipids and membrane fluidity, yet lipids and fluidity are also suggested to change in response to fluid shear stress and potentially mediate its sensing.^[^ ^126^, ^129^ ^]^ How do the PIEZOs and lipids relate to each other in time and space when shear stress changes? Are changes in PIEZOs a driver of changes in lipids?

### 8.5 Vascular Diversity

Vasculatures are vast complex structures comprising many features formed and regulated by plastic cell phenotypes across organs and life courses. The current thinking is that PIEZOs enable force sensing in all vascular biology. If this is the case, how are PIEZOs selected, used and adapted for different vascular contexts such as arteries, capillaries, lymphatics and veins, and diverse body parts, organs and tissues such as aorta, bone, brain, eye, heart, intestine, kidney, liver, lower limb, lung, skeletal muscle, penis, placenta, skin, tumor and uterus? Comprehensive vascular maps of PIEZO mRNA, protein, protein localization, channel properties and regulators would likely help to provide some answers. The maps would almost certainly need to be integrated with information about other molecular systems, such as other regulators of intracellular Ca^2+^ and membrane potential, because PIEZOs are not alone; PIEZOs may be minor contributors if other ionic mechanisms drown them out electrically or downstream pathways are limited, or they may conversely be major contributors if other ionic mechanisms are suppressed, or downstream pathways amplified. A generic map of all factors could be a template for refinements according to sex, age, species and other contexts. Computational approaches are again likely to be increasingly necessary to handle and interpret the large complex data sets. The scale of the technical challenge is illustrated by preliminary transcript information across cell and tissue types (https://www.gtexportal.org/home/gene/PIEZO1, https://www.gtexportal.org/home/gene/PIEZO2) that falls short of the maps that would be needed to understand PIEZO vascular biology.

### 8.6 Pathophysiology and Therapeutics

Are PIEZOs important therapeutic targets and, if so, in which disease conditions specifically (Tables 1 and 2)? Is it possible to develop selective PIEZO modulators that are safe to administer to people? Could more detailed molecular understanding of the channels enable predictions of effects of genetic variants on these proteins, thus potentially improving patient care?

The rapidly expanding evidence for targeting PIEZOs in unsolved human disease problems (Tables 1 and 2) is motivating for new drug discovery efforts. Nevertheless, there are obstacles ahead for researchers seeking patient benefits. One obstacle is the sometimes‐diverging conclusion about whether a PIEZO activator or inhibitor would be therapeutically advantageous (Tables 1 and 2), and whether an activator or inhibitor would potentially induce unacceptable adverse effects despite therapeutic benefits arising. Another obstacle is the successful expansion of a PIEZO pharmacology toolkit coupled with understanding of the structure‐activities and physiochemical properties of the pharmacological modulators as well as determinations of the molecular details of the modulator binding sites on the target protein (e.g., PIEZO or an associated partner protein).

## 9 Conclusions

There has, therefore, been an explosion of new knowledge suggesting wide‐ranging significance of PIEZOs across vascular biology from the embryonic to the adult stages in health, disease and old age. These intriguing, complex membrane proteins seem to act solely by conferring high fidelity, fast, robust mechanical force sensing coupled with efficient transduction of the sensed force into downstream effects through transmembrane ion flux. They seem to exist solely to enable cells to know their mechanical context and respond appropriately to changes in it. The dominant PIEZO of endothelium, PIEZO1, is pivotal in a central defining feature of the cardiovascular system that is its ability to detect fluid flow and thereby match vascular and valvular architectures to tissue needs. PIEZO1 is by no means exclusive to endothelium however, as it is expressed also in other cell types such as vascular smooth muscle cells that importantly sense forces such as tension from vessel wall stretch. Indeed, most if not all vascular cell types may take advantage of the benefits conferred by PIEZO1. There is, for example, also PIEZO1 expression in perivascular fat cells.^[^ ^244^, ^245^, ^246^ ^]^ Moreover, despite PIEZO1's primary roles in the vasculature, PIEZO2 also contributes, most obviously in perivascular neurons but also other contexts such as endothelium.

Key concepts are emerging, again best evidenced so far from studies of PIEZO1. These channels behave as adaptable force sensing units plugged into diverse vascular cell types, structures and contexts, capable of sensing apparently different forces and triggering different outcomes such as vascular expansion and arrest depending on the amplitude and type of force. The channels are referred to in this article as force‐sensing e‐transducers to emphasize their ability to both feel force and take advantage of powerful preexisting ionic gradients for quick and efficient transduction of channel activation into cell and tissue effects (Figure 1). Numerous molecular mechanisms are becoming apparent for regulating the channels and adapting them to different situations. A key regulated property is their inactivation, which is a type of molecular memory that may enable cell responses to depend on the prior mechanical experiences of the cells. Ca^2+^ entry is also crucial, entering through the channels to be the pivotal signal orchestrating downstream events for appropriate cell and tissue changes (Figure 2). The specific roles of these channels and their importance may depend strongly on animal behaviors such as physical activity and life stages such as old age. These are just a few examples of the new knowledge covered in more depth in this article. We can expect in the future further exciting PIEZO knowledge, PIEZO concepts, challenges to PIEZO ideas and hopefully PIEZO‐targeted medicines.

## Conflict of Interest

The author is a partner of CalTIC GmbH, a pharmaceutical start‐up company with a mission to develop a new class of medicines for the treatment of cardiovascular and metabolic diseases.

## Data Availability

Data sharing is not applicable to this article as no new data were created or analyzed in this study.
